# Oxidative Stress in Age-Related Neurodegenerative Diseases: An Overview of Recent Tools and Findings

**DOI:** 10.3390/antiox12010131

**Published:** 2023-01-05

**Authors:** Dimitris Korovesis, Teresa Rubio-Tomás, Nektarios Tavernarakis

**Affiliations:** 1 Institute of Molecular Biology and Biotechnology, Foundation for Research and Technology—Hellas, GR-70013 Heraklion, Greece; 2 Division of Basic Sciences, School of Medicine, University of Crete, GR-71003 Heraklion, Greece

**Keywords:** ageing, age-related pathology, neurodegenerative disease, oxidative stress, protein aggregation, reactive oxygen species (ROS), small molecule probes

## Abstract

Reactive oxygen species (ROS) have been described to induce a broad range of redox-dependent signaling reactions in physiological conditions. Nevertheless, an excessive accumulation of ROS leads to oxidative stress, which was traditionally considered as detrimental for cells and organisms, due to the oxidative damage they cause to biomolecules. During ageing, elevated ROS levels result in the accumulation of damaged proteins, which may exhibit altered enzymatic function or physical properties (e.g., aggregation propensity). Emerging evidence also highlights the relationship between oxidative stress and age-related pathologies, such as protein misfolding-based neurodegenerative diseases (e.g., Parkinson’s (PD), Alzheimer’s (AD) and Huntington’s (HD) diseases). In this review we aim to introduce the role of oxidative stress in physiology and pathology and then focus on the state-of-the-art techniques available to detect and quantify ROS and oxidized proteins in live cells and in vivo, providing a guide to those aiming to characterize the role of oxidative stress in ageing and neurodegenerative diseases. Lastly, we discuss recently published data on the role of oxidative stress in neurological disorders.

## 1. Introduction

**Figure 1:**
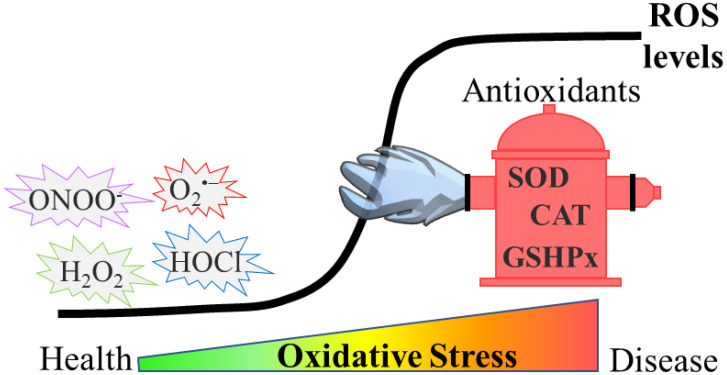
Development of oxidative stress. In physiological conditions, ROS remain at low levels by the effect of antioxidants such as superoxide dismutase (SOD), catalase (CAT) and glutathione peroxidase (GSHPx). However, during oxidative stress, ROS levels rise, due to either higher production or impaired antioxidant defense (or both), and this results in the accumulation of damaged biomolecules. Such events are detrimental and either drive or exacerbate diseases such as neurodegenerative disorders.

Oxidative stress can be defined as an imbalance between the generation of reactive oxygen species (ROS) and their quenching by antioxidants in a specific cell or tissue (Figure 1). One of the most studied ROS is hydrogen peroxide (H_2_O_2_), but many other, more reactive species, such as superoxide anion radicals (O_2_^•−^), hydroxyl radicals (^●^OH), singlet oxygen (^1^O_2_), nitrogen dioxide (NO_2_^●−^), hypochlorous acid (HOCl) and peroxynitrite (ONOO^−^) are generated by various enzymatic and non-enzymatic processes in biological systems [1].

The excessive amount of ROS triggers the so-called redox-dependent signaling in physiological conditions. However, when it surpasses a threshold, ROS become detrimental. Apart from aberrant redox signaling, ROS can damage a myriad of cellular components, such as proteins, DNA and lipids. As several amino acid residues are amenable to oxidation (depending on the ROS involved), oxidative damage leads to a variety of oxidative modifications onto proteins. These modifications could alter cellular function by regulating stability, activity, subcellular localization or the protein–protein interaction of oxidized proteins [2]. A wide range of cellular mechanisms are activated to eliminate ROS as well as the ROS-mediated oxidized biomolecules (such as the newly discovered protection of promoters from oxidative damage though the action of the nuclear mitotic apparatus protein, NuMA [3]). Nonetheless, upon persistent activation these mechanisms may either aggravate the cellular damage or become impaired, eventually leading to a vicious cycle that promotes the progression of several pathologies.

Oxidative stress is implicated in a wide variety of age-related diseases ranging from diabetes and cardiovascular disease to cancer and neurodegenerative diseases [4,5,6]. A common characteristic of most neurodegenerative diseases is the formation of toxic protein aggregates that affect many functions, eventually leading to neuronal loss. Interestingly, several of these proteins can become oxidized. While this may not come as a surprise since protein oxidative damage is a non-discriminative process, studies on α- synuclein [7], prion protein [8] and TDP-43 [9] have shown that their oxidation is in fact required for their initial nucleation to take place. This may indicate that the oxidation of aggregation-prone proteins may constitute another common characteristic among neurological disorders. The role of oxidative stress in these diseases is further evident by the fact that oxidative damage has been consistently detected in the post-mortem brains of patients with Alzheimer’s (AD), Parkinson’s (PD) and Huntington’s (HD) diseases [10]. While it is currently unknown whether oxidative stress is a driver or a consequence that further exacerbates them, these findings point to the dependency of disease progression on oxidative stress.

All the above highlight the need for further insight into the interplay between oxidative stress and neurodegenerative diseases. Several methods for assessing oxidative stress have been developed over the years. Beyond traditional (e.g., electron spin resonance (EPR)-based techniques and mass spectrometry [11]) and commercial assays, these methods include novel tools for selectively detecting ROS and achieving global profiling of damaged proteins in live cells or organisms. While more reliable and informative, the exploitation of these tools is extremely limited and, when utilized, is often in fields other than neurodegenerative diseases. Hence, this review focuses on presenting both recent advanced tools for evaluating oxidative stress and findings that emphasize how oxidative stress may contribute to the progression of neurodegenerative disorders.

## 2. Protein Aggregation

**Figure 2:**
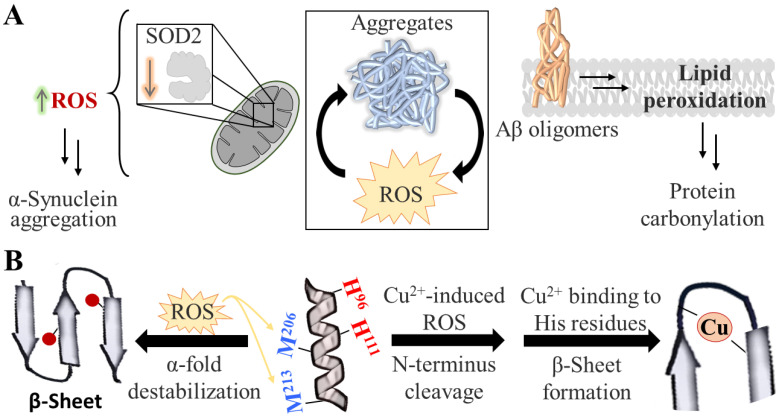
Interplay between protein aggregates and oxidative damage. (**A**) Increased protein aggregation in several neurodegenerative diseases is associated with increased ROS levels. This relationship is bidirectional; excessive ROS production is found to induce protein aggregation and vice versa. Two examples of this vicious cycle are shown: (1) a decrease in the mitochondrial levels of Superoxide dismutase 2 (SOD2) gives rise to elevated ROS, which in turn promotes A-synuclein aggregation (right panel) and (2) the accumulation of Aβ oligomers induces lipid peroxidation, an oxidative damage that further results in protein carbonylation (left panel). (**B**) Proposed mechanisms of ROS-induced aggregation of prion protein. At physiological conditions, part of the prion protein exists in the α-fold form (depicted as a helix in the middle). However, in disease, its scrapie isoform forms β-sheets, which eventually drive fibrillation. The transition from α-fold to β-sheet is proposed to be mediated by ROS, with two different mechanisms: (1) by methionine oxidation (depicted as red circles), which destabilizes the α-fold form (left side) or (2) by ROS-mediated cleavage—induced by copper—of the protective N-terminus of prion, and subsequently, β-sheet formation due to copper chelation by neighboring histidine residues. The position of the residues shown is for illustration only and do not correspond to their true location.

Protein aggregation constitutes an example of this vicious cycle of negative effects, and it plays a key role in the pathology of neurodegenerative diseases. In the case of PD, AD and HD, the presence of aggregates of α-synuclein, Aβ peptide, and Huntingtin with polyglutamine expansion, respectively, alone or together with other known, or yet-to-be-discovered peptides, has been linked to the symptomatology and progression of the diseases [12,13,14]. Compelling data suggest that the relationship between oxidative stress and protein aggregation is bidirectional, since aggregation-prone conformers exacerbate the production of ROS (e.g., Aβ has been shown to initiate lipid peroxidation [15]), whereas, in turn, oxidative stress promotes protein aggregation (Figure 2A). While molecular-level mechanistic details of the latter route are very sparse, in silico studies on prion protein have suggested that ROS could induce *β*-sheet formation (a form prone to aggregation) either directly or indirectly. In the first case, this is caused by destabilization of prion’s physiological *α*-fold form due to the direct oxidation of methionine residues by ROS (Figure 2B, left) [16]. In the second case, ROS (generated by copper reduction) initially induce cleavage of prion’s N-terminus and, subsequently, the remaining copper promotes β-sheet formation due to its chelation by neighboring histidine residues (Figure 2B, right) [17]. While these mechanisms need to be validated further and may not be representative for other disordered proteins, they certainly exemplify the complex relationship between oxidative stress and protein aggregation.

In line with the above, it has been proposed that, during ageing, age-related damage in the brain activates stress response pathways that rely on redox signaling through the production of H_2_O_2_ as a second messenger. It is widely accepted that this occurs as an attempt to restore homeostasis. Over time, the accumulation of damage leads to ROS concentrations that exceed the physiological levels required for activating signaling pathways. Hence, ROS shifts from its beneficial stress response-associated effects to its detrimental outcomes due to ROS-dependent damage through a vicious cycle that inevitably results in high ROS levels [10]. According to this hypothesis, damage due to the formation of aggregates, such as Aβ and tau oligomers in AD [18,19], can induce ROS generation. While this has been shown for different types of aggregates, it is still debatable which aggregating species are responsible for inducing ROS generation. A study in induced pluripotent stem cells showed that both oligomeric and fibrillar α-synuclein were able to increase ROS levels [20]. However, another study in yeast using Aβ42 peptide variants with different aggregation propensity found that peptides that are unable to be recruited into inclusions induce ROS regeneration, while insoluble deposits do not [21]. Collectively, all the above underline the strong relationship between oxidative stress and protein aggregation in neurodegenerative diseases.

## 3. ROS Generation and ROS-Related Processes

Oxidative stress is mediated by several exogenous (such as pathogens, dietary components, toxic substances, pharmaceutical drugs) and endogenous factors. With respect to endogenous pro-oxidant events, broadly speaking, this includes lowering the antioxidant defense (e.g., downregulation enzymatic or non-enzymatic antioxidants or loss-of-function mutations of enzymes) or inducing ROS generation and propagation (e.g., through the activation of mitochondrial NADPH oxidase). The dysfunction of mitochondria and peroxisomes (often interlinked), increase in free metal ions, as well as ROS themselves (through oxidative damage), could further promote oxidative stress. In the next sections we will focus on the relation between ROS and mitochondria, iron signaling and parthanatos.

### 3.1. Major ROS Sources

Within cells, mitochondria produce ROS which are involved in both physiological signaling and the oxidative damage of different cell components, including the mitochondria itself. O_2_^•−^, the primal ROS produced in mitochondria, is generated by the one-electron reduction in molecular oxygen (O_2_) during oxidative phosphorylation for the synthesis of ATP. However, mitochondria also generate H_2_O_2_, which is produced by the dismutation of O_2_^•−^ catalyzed by mitochondrial SOD. Thus, diffusion-restricted O_2_^•−^ derived from the electron transport chain (ETC) can be converted by SOD enzymes (especially SOD2) into highly diffusible secondary messenger H_2_O_2_ [22]. Redox cofactors such as NADH are essential, both to combat oxidative stress and to facilitate reductive reactions in biosynthesis (for example, proline biosynthesis). The amounts and ratios of oxidized and reduced NAD(H) and NADP(H) affect biosynthetic capability and, subsequently, regulate cell proliferation [23,24,25]. While mitochondria are vital to the cell survival, aberrant mitochondrial homeostasis (the so-called dysfunctional mitochondrial quality control, including dysregulated mitophagy) has been implicated in the pathogenesis of neurodegenerative diseases, such as AD [26], PD [27] and HD [28], and it has, thus, been proposed as a promising therapeutic target [29].

In addition to mitochondria, other sources of ROS exist inside the cell. Peroxisomes are organelles that participate in metabolic oxidative processes (β-oxidation of very-long-chain fatty acids, α-oxidation of branched-chain fatty acids, oxidation of D-amino acids and polyamines), as well as the synthesis of ether phospholipids, bile acids and docosahexaenoic acid. These processes produce ROS as byproducts, which are subsequently neutralized by antioxidant enzymes, including catalases. Peroxisomes crosstalk with the nucleus, mitochondria, endoplasmic reticulum (ER) and lysosomes, and these inter-organelle communications are key to maintain peroxisome function. Of note, peroxisomal dysfunction has been described in disease and during ageing and is associated with many cellular alterations, highlighting its important roles in redox homeostasis [30].

### 3.2. Ferroptosis

Iron is an important signaling molecule that is related to oxidative stress (for instance, it is required, along with H_2_O_2_, for the generation of ^●^OH) and neurodegenerative disease. Consequently, the brains of patients with AD and PD contain high iron concentrations [31], and mechanistical studies highlight the importance of iron homeostasis for proper neuronal function, since both iron deficiency [32] and overload [33] negatively impact neuronal function.

Ferroptosis is an iron-dependent type of programmed cell death that involves oxidative stress, since it is accompanied by lipid peroxidation [34]. It has been found that α-synuclein aggregation drives ferroptosis via aggregate–membrane interaction in human stem cell-derived models of synucleinopathy. Importantly, α-synuclein oligomers further induce lipid peroxidation, and the inhibition of lipid peroxidation blocks the aggregate–membrane interaction, indicating that the oxidation state of the lipid membrane is key for triggering ferroptosis in this experimental model of PD [35]. Therefore, stress-induced lipid peroxidation could promote ferroptosis and its accompanying neurodegeneration. Importantly, there is a complex interplay between iron regulation (and, subsequently, ferroptosis) and the alteration of mitochondrial homeostasis in a plethora of neurodegenerative diseases, including AD and PD [36,37,38]. The mechanisms underlying this crosstalk are under study. For instance, an in vivo study using peroxiredoxin 5 (Prx5)-knock out mice showed that Prx5 deficiency-induced iron overload causes neuronal toxicity and eventually activates neuronal death in the hippocampus through endoplasmic reticulum-mediated mitochondrial fission [39].

Numerous molecular mediators of ferroptosis have been suggested as pharmaceutical targets, including nuclear factor erythroid 2-related factor-antioxidant response element (Nrf2-ARE), n-acetylcysteine (NAC), Fe^2+^, NADPH, and its oxidases NOX, among others [40], underlying its significance in pathological conditions.

### 3.3. Parthanatos

Parthanatos is a programmed cell death signaling pathway in which excessive oxidative damage to DNA by ROS (e.g., peroxynitrite (ONOO^−^), among others) rapidly hyperactivates poly(ADP-ribose) polymerase (PARP). Hyperactivated PARP induces the downstream formation of large poly(ADP-ribose) polymers that subsequently promote the release of apoptosis-inducing factor (AIF) from the outer mitochondrial membrane into the cytosol, where it forms a complex with macrophage migration inhibitory factor (MIF). The complex translocates into the nucleus, where it triggers DNA degradation and cell death. Parthanatos underlies a wide spectrum of diseases, including those affecting the nervous system, such as AD, PD and stroke [41,42].

## 4. Recent Developments for Evaluating Oxidative Stress

During oxidative stress, high levels of ROS result in damage to proteins, nucleic acids and lipids [6]. Due to its implication in several diseases, the development of methods for evaluating oxidative stress has been an important aspect in the field. Oxidative stress can be assessed by determining ROS levels, the oxidative damage or the antioxidant status. Methods for measuring ROS [5,11], oxidized biomolecules (proteins [43], lipids [44,45] and nucleic acids [46,47]) and antioxidant status [48,49] have been previously reviewed and, importantly, good practice guidelines for such measurements were very recently set out by leading experts in the field [4]. Herein, we focus on recent and notable developments and aim not only to inform the readers, but also to inspire further research into the field. We limit our discussion to methods for both the direct measurements of ROS and the oxidative damage (indirect effect) on proteins. All tools, along with their application method and their key features, reported in this section are summarized in Table 1 to assist the reader in locating the most suitable tool for their purpose.

### 4.1. Direct Probing of ROS

To understand whether oxidative stress is implicated in a certain condition or disease, it is crucial to define ROS levels. A variety of ROS are produced either by natural cellular processes or by the reactions of ROS themselves with other molecules (e.g., hypohalous acids are generated by the enzymatic peroxidation of halogen ions with H_2_O_2_ [71]). Deciphering which particular ROS is in excess in a certain disease background could offer an insight into the cellular mechanism involved. While the reaction rates of the different ROS vary (e.g., radical vs. non-radical ROS), ROS have overlapping chemical properties, rendering their specific identification challenging. As such, great effort has been made to develop small molecule probes and genetically encoded reporters for discriminating ROS within the cellular milieu. Since fluorescence microscopy is the preferred method in most biological experiments (for both live cell or in vivo samples), in this section we focus on fluorescence imaging tools, as they can be used together with other fluorescently labeled molecules or markers.

#### 4.1.1. Hypohalous Acids (HOX)

HOX are ROS which are produced by peroxidases in the presence of H_2_O_2_ and any of chloride, bromide or thiocyanate (a pseudohalide). As opposed to hypothiocyanous acid (HOSCN), HOCl and hypobromous (HOBr) acids are strong oxidants and less selective [4]. Among the three, HOCl has attracted a lot more attention as ROS since chloride plasma concentration is much higher compared to the others [72].

**Figure 3:**
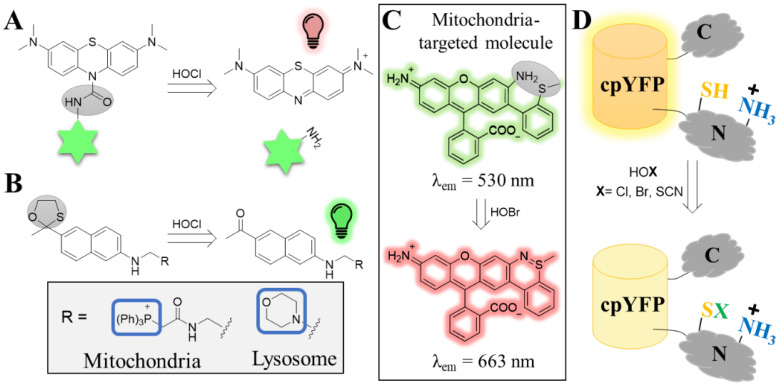
Tools for the visualization of hypohalous acids. (**A**) Structure and turn-on mechanism of the methylene blue-based probe (FDOCl-18) upon sensing HOCl. The methylene blue (top structure) is conjugated to naphthalene (indicated as a green star) through the HOCl-sensing group (highlighted by the gray oval, left structure). The methylene blue is non-fluorescent while the sensing group is attached. Upon sensing HOCl, a deformylation mechanism takes place that leads to the liberation of the methylene blue, which is now fluorescent. (**B**) Sensing mechanism of the organelle-specific turn-on probe for HOCl. The fluorescence properties of a two-photon dye (acedan) were quenched by HOCl-labile group (gray circle, left structure). The probe was further equipped with organelle specific-targeted moieties to drive it either to mitochondria (MITO-TP) or to lysosomes (LYSO-TP). (**C**) Structure of the HOBr ratiometric probe RhSN-mito. In the absence of HOBr, the probe emits at 530 nm. Reaction of the sensing moiety (gray oval, top structure) with HOBr leads to the formation of an additional ring that alters the fluorescence properties of the probe, which now emits at 663 nm. (**D**) The NemR-cpYFP construct (Hypocrates) for detecting hypohalous acids. The YFP was integrated onto a flexible loop of the NemR. While the YFP exhibits the high fluorescence in non-oxidation conditions, the intensity decreases when the thiol of the cysteine senses the hypohalous acid.

Despite the variety of previously reported probes [73], HOCl probes with near-infrared (NIR) emission and high sensitivity (i.e., at low nanomolar range), both desired properties for in vivo application, were missing. To this end, Yi and co-workers reported the development of FDOCl-1, a turn-on probe with excellent selectivity and high sensitivity to HOCl. The probe was successfully used to monitor HOCl production in the joints of inflammation on a mouse model of arthritis [50]. The team subsequently coupled the probe onto a green-emitting naphthalene (generating probe FDOCl-18) to enable visualization of the probe distribution in the “off” mode [51]. Upon encountering HOCl, the naphthalene falls off and the NIR dye turns on (Figure 3A).

Due to their high reactivity and low permeability, some ROS do not escape the site of their production [5,74]. This is why it is highly desirable to have tools to track ROS at the subcellular level. HOCl is one such ROS as it reacts rapidly with nearby thiolates [75]. Chang and co-workers therefore developed turn-on probes that enable monitoring HOCl distribution at specific organelles [52]. The probes were constructed by combining a two-photon dye with chemical moieties that target either the mitochondria or the lysosomes (termed, MITO-TP and LYSO-TP, respectively, Figure 3B). The probes were successfully applied on inflamed murine models and showed that both the mitochondria and lysosomes of macrophage cells generate higher levels of HOCl during inflammation.

While many HOCl-sensing probes have been shown to be selective against other ROS, selectivity is not usually assessed against HOBr or HOSCN. This may imply that such HOCl probes cannot discriminate between the different hypohalous acids. This is not the case for the HOBr-sensing probes reported by Tao and co-workers. This ratiometric probe, namely RhSN-mito, was initially developed based on a previous observation that HOBr, but not HOCl, crosslinks the sulfur of methionine with proximal amines on collagen IV [76]. The probe was shown to be selective for HOBr against many ROS, including HOCl, and suitable for in vivo imaging in zebrafish [77]. The authors later reported a derivative of the first probe, which now exhibited NIR emission upon encountering HOBr, an improvement better suited for in vivo application (Figure 3C) [53].

Genetically encoded fluorescent reporters for monitoring ROS are perhaps more often the method of choice, and such tools have offered valuable insight in the field. However, the development of such reporters for hypohalous acids was considered very challenging. This is because many ROS reporters rely on the oxidation of cysteines to modify their fluorescent properties (either switch on or off). While hypohalous acids react rapidly with cysteines, selectivity is hard to be achieved as cysteines are susceptible to oxidation by many other ROS. In order to develop a HOX-selective biosensor, Belousov and co-workers utilized the *E. coli*-derived transcription repressor NemR [54], a protein that had previously been shown to contain a certain cysteine (Cys106) prone to oxidation by chlorine species [78]. The team therefore developed the so-called Hypocrates sensor by integrating a YFP protein between the DNA-binding and the sensory domain of NemR, the latter containing Cys106 (Figure 3D). The reporter was shown to be sensitive to all three hypohalous acids with unexpectedly very good selectivity over other ROS. The sensor was successfully applied to live cells and in vivo.

#### 4.1.2. Superoxide Anion (O_2_^•−^)

While not very reactive, O_2_^•−^ is one of the most important ROS; not only is it generated by two major ROS sources (ETC [79] and NADPH [1]), it is also a precursor of other ROS such as H_2_O_2_ and ONOO^−^ [6]. Despite its importance, traditional methods for measuring O_2_^•−^ have certain limitations and may not provide accurate measurements, as recently outlined [4]. For instance, while the circularly permuted yellow fluorescent protein (cpYFP) was initially reported as an O_2_^•−^ sensor [79], it was later shown to be completely unresponsive to it [80], making it imperative that more reliable tools are developed. Hua and co-workers reported the development of DMPS-O, a turn-on small molecule probe that exhibited excellent selectivity and detection limit at low nanomolar range [55]. DMPS-O has NIR emission, making it suitable for in vivo imaging, and, importantly, a mitochondria-targeting group that enables measuring O_2_^•−^ at its production site, a crucial aspect given that it is short-lived. While it was not assessed in any living organism, such probes could be used in in vivo systems, as shown by Liu and co-workers, who developed a turn-on probe, namely HQ, using a dye with two-photon excitation that minimizes damage and has better penetration [56]. The suitability of HQ was demonstrated in a lung inflammation mouse model to monitor endogenous O_2_^•−^ fluxes. Lastly, an interesting concept for monitoring ROS production at the protein level was shown by Chen and co-workers [57]. Monoamine oxidase B (MAO-B) is a mitochondrial-localized enzyme that generates ROS as a by-product through the oxidation of biogenic amines. MAO-B has attracted considerable interest, for it is found to be elevated during aging as well as in several neurodegenerative disorders. To facilitate the detection of ROS production by MAO-B, the authors developed a ratiometric cyanine-based probe (termed MitoHCy-NH_2_) equipped with a MAO-B- and a mitochondria-targeting group. They successfully showed that their probe can monitor activity and ROS generation by MAO-B in cells and in an aging mouse model.

#### 4.1.3. Peroxynitrite (ONOO^−^)

As mentioned earlier, ONOO^−^ is generated by the reaction of O_2_^•−^ with nitric oxide (NO^•^). Although the reaction is not catalyzed by any enzyme (in contrast to ROS already discussed), ONOO^−^ production is likely restricted to sites where O_2_^•−^ is present, since the latter is short-lived and impermeable [81]. ONOO^−^ is highly reactive and apart from causing oxidative damage (e.g., tyrosine nitration and lipid peroxidation), it is also responsible for generating other reactive species such as nitrogen dioxide, carbonate and radicals [59]. Tools that selectively detect ONOO^−^ over other reactive ROS could contribute to deepening our understanding of its role in health and disease.

**Figure 4:**
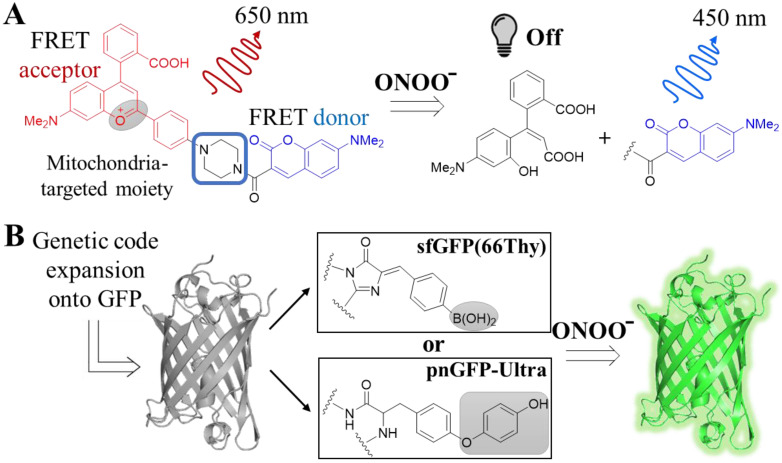
Peroxynitrite-selective tools and their mechanism of detection. (**A**) Structure and mechanism of the small molecule FRET probe MITO-CC. The probe consists of a FRET donor and an acceptor as well as a linker, which is also the group responsible for sending the probe into mitochondria. In the absence of ONOO^−^, the probe emits at 650 nm due to energy transfer from the donor to the acceptor. However, upon reaction with ONOO^−^, the FRET acceptor is eliminated and decomposed, and the remaining donor fluoresces at 450 nm. (**B**) GFP variants sfGFP(66Thy) and pnGFP-Ultra with non-natural amino acids. Genetic code expansions on GFP using non-natural amino acids (shown in the boxes) led to the generation of GFPs that are non-fluorescent. The non-natural amino acids are equipped with ONOO^−^ sensitive groups, which upon reaction are cleaved off and the resulting GFP is then fluorescent.

Various small molecule probes, equipped with different chemical groups for sensing ONOO^−^, have been developed over the years. However, selectivity was in many cases an issue, since those groups would also be targeted by other ROS, given the low in cellulo concentrations of ONOO^−^ compared to other ROS [82]. Recent efforts have yielded exceptionally selective turn-on [82,83] or -off [84] probes, with additional desirable features for either in vivo application, such as NIR emission [83], two-photon excitability [82] or organelle-specific detection (e.g., lysosomal localization [84]). A further improvement of the above examples constitutes the probe developed by Chang and co-workers. The team reported the development of MITO-CC, a FRET-based probe with two-photon excitability that can selectively detect ONOO^−^ in mitochondria [58]. MITO-CC emits at the NIR spectrum in the absence of ONOO^−^ but at the UV-vis range upon its detection, as the FRET acceptor falls off and decomposes (Figure 4A). The authors demonstrated that the probe could detect endogenous ONOO^−^ in both live cells and a mouse inflammation model.

Enriching the tools available for the selective detection of ONOO^−^ with genetically encoded reporters is equally challenging, as in the case of HOX-sensing reporters. A way to overcome the limitations related to protein manipulations permitted by traditional approaches (i.e., mutations) is by genetic code expansion [85], as exemplified by two studies. In an attempt to develop fluorescent protein reporters for ONOO^−^, two independent teams employed genetic code expansion to create GFPs with non-canonical amino acids, namely sfGFP(66Thy) and pnGFP-Ultra, respectively [59,60]. The non-canonical amino acids used were tyrosine mimics, and they were chosen to contain peroxynitrite-labile moieties. Genetic code expansion enabled them to replace the tyrosine that is part of GFP’s chromophore with their non-canonical amino acids so that the protein is not fluorescent. Upon reaction with ONOO^−^, but not with other ROS, the moieties fall off and GFP turns on (Figure 4B). While none of the studies used their reporters in an in vivo system, genetic code expansion can be applied in animals [86].

#### 4.1.4. Hydrogen Peroxide (H_2_O_2_)

H_2_O_2_ is a non-radical ROS that is mainly produced by superoxide dismutase (SOD) through the dismutation of O_2_^•−^. Despite its low reactivity, H_2_O_2_ is a key player in oxidative stress since it can not only modify cysteines on certain proteins, but also because it is a precursor of the highly reactive hydroxyl radical. Out of a large variety of tools available for H_2_O_2_ detection, herein we discuss two examples, one small molecule probe and one protein reporter, that offer exceptional advantages over previous tools. It should be noted that the low reactive nature of H_2_O_2_ means that tool selectivity cannot be high, and thus, additional control experiments should be performed to dissect the contribution of other ROS.

**Figure 5:**
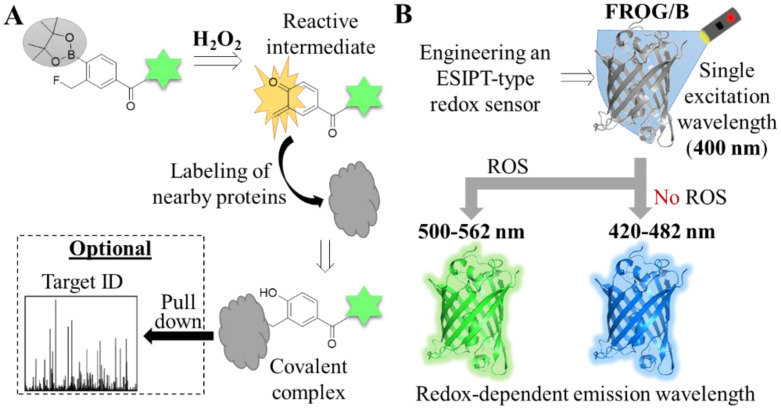
Tools for detecting H_2_O_2_. (**A**) Structure and mechanism of H_2_O_2_-responsive protein labeling using Hyp-L. A fluorescently labeled (indicated as a green star) small molecule probe with a H_2_O_2_-labile group (gray circle) forms a reactive intermediate upon oxidation, which is in turn inserted onto proximal proteins. The proteins can be visualized by fluorescent microscopy or subjected to proteomics workflow for identification. (**B**) Mechanism of the excitation state intramolecular proton transfer (ESIPT)-based protein reporter. A GFP was engineered to develop an ESIPT-based protein reporter (FROG/B). The final variant is excited by a single wavelength, but the emission wavelength can change depending on the presence or absence of ROS.

As discussed earlier, ROS probes could be designed to target some specific organelles (i.e., mitochondria or lysosome). While useful, this approach does not allow the indiscriminate characterization of which organelles or cellular processes could, for instance, induce ROS generation. Hamachi and co-workers reported a novel approach to fill this gap. The authors carefully designed Hyp-L, a small molecule probe that, upon reacting with H_2_O_2_, forms an intermediate that rapidly reacts with proximal proteins, forming covalent adducts (Figure 5A) [61]. Hyp-L was equipped with a dye (for which a commercial antibody exists) to allow imaging and, importantly, pull-down experiments of the probe-tagged proteins. With this H_2_O_2_-responsive protein labeling, they were able to profile the H_2_O_2_-surrounding proteome and show that activated RAW264.7 macrophages, autophagosomes, as well as phagosomes, endosomes and lysosomes, are rich in H_2_O_2_.

Two significant benefits of ratiometric imaging are the monitoring of the distribution of the probe that is responsive to a certain stimulus (and hence, it does not emit light in its absence or presence) and allowing accurate quantitative analysis. In the field of protein reporters for ROS, this has been achieved either by the use of a FRET pair or ratiometric probes. Both types, however, have some limitations, namely, the size of the construct (for FRET pair probes), degradation or time lag of maturation of one of the FRET pair proteins, or the use of two excitation lasers (for ratiometric probes). To enable ratiometric imaging free of the aforementioned limitations, Hisabori and co-workers developed a ROS reporter that uses the excitation state intramolecular proton transfer (ESIPT) mechanism [62]. ESIPT-based reporters are constituted by one fluorescent protein that, despite being excited with a single wavelength, emits light on two different wavelengths, depending on the stimulus. Following several rounds of mutations, the authors successfully engineered a GFP sensor (termed FROG/B) which constitutes the first example of an ESIPT-based protein reporter for detecting ROS (Figure 5B). The authors used FROG/B to visualize redox changes in cyanobacterial cells.

### 4.2. Mapping Protein Oxidative Damage

**Figure 6:**
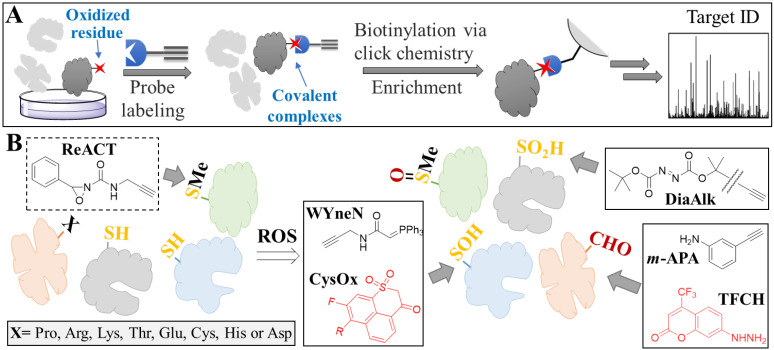
Chemical proteomics-based identification of oxidized proteins. (**A**) General workflow of chemical proteomics. The proteome of interest consisting of non-oxidized (indicated as light gray) and oxidized (indicated as dark gray. Oxidized residue is depicted as a red star) proteins is treated with a probe specific for an oxidized amino acid. Upon reaction with the target protein, a covalent complex is formed. Following cell lysis, the probe-modified proteins are biotinylated (using click chemistry) and enriched with streptavidin beads. Finally, digestion and analysis by LC-MS/MS leads to target identification. (**B**) Tagging of proteins with small molecule probes. Upon oxidation with ROS, amino acid residues are modified. The structures of the probes targeting different amino acids, suitable either for imaging (red structures) or chemical proteomics, are shown in boxes. Gray arrows show which probes react with which amino acids.

While characterizing which ROS is elevated, at which organelles and to what extent, it is often challenging to obtain the full picture of events. Apart from potential issues associated with probe selectivity, distribution or stability, this is because of the intrinsic reactivity of ROS, which renders them difficult to be detected before reacting with a wealth of surrounding biomolecules (unless extremely high probe concentration is used). Assessing oxidative stress by looking downstream of ROS production (e.g., protein oxidative damage) largely overcomes these issues. Understanding the oxidative damage of proteins may also offer additional insight regarding protein function (e.g., inhibition [87] or activation [88] upon ROS-mediated oxidation). In this section, we present recent applications of small molecule probes for visualizing or identifying oxidized proteins. For the latter, we focus on probes for mass spectrometry (MS)-based chemical proteomics [89], the general workflow of which is shown in Figure 6A.

#### 4.2.1. Oxidized Methionine

Methionine is a sulfur-containing amino acid and, as such, it can first be oxidized to sulfoxide and subsequently to sulfone. Oxidized methionine has been shown to play a pivotal role in strengthening protein–protein interactions [90] or promoting protein aggregation [8], and so it is essential to develop methods for identifying proteins with such oxidative forms. Unfortunately, no probes have been reported so far to target either of these forms. In order to bypass the need for oxidized methionine-targeted probes, Chang and co-workers recently developed the first ever selective probe targeting non-oxidized methionine (ReACT, Figure 6B) [63], which had previously not been possible due to the weak nucleophilicity of methionine. The idea behind their approach was that oxidized methionine-containing proteins could be indirectly identified if the pool of probe-bound proteins between control (no ROS) and sample (with ROS) were compared (since ReACT can facilitate the MS-based identification of methionine-containing proteins). Application of this workflow recently demonstrated the power of this tool, as it revealed that pancreatic cancer metastasis is driven by methionine oxidation-mediated activation of pyruvate kinase M2 [88].

#### 4.2.2. Oxidized Cysteine

Owing to its high reactivity among amino acids, cysteines (catalytic or not) react rapidly with ROS and become oxidized. Upon oxidation, the thiol group can form disulfide bonds, become nitrosylated, or be converted to sulfenic, sulfinic and sulfonic acids. Herein, we focus only on probes that directly recognize certain modifications (i.e., sulfenic and sulfinic acids), but it should be noted that it is possible to extract information for a certain cysteine modification for which no specific probe exists, as discussed for methionine. For instance, Zahedi and co-workers reported a workflow for specifically isolating proteins that had undergone S-nitrosylation [91].

Most reported probes for both the imaging and profiling of sulfenic or sulfinic acid-modified proteins have been developed by the Carrol lab. Due to cysteine’s reactivity, the difficulty here is to develop probes that react with one oxidized form but not with cysteine. Following a comparative analysis of several newly synthesized probes, Carrol and co-workers designed a probe, termed BTD, which exhibited great reactivity, as demonstrated by the identification of over 700 sulfenic acid-modified proteins against a colon cancer cell proteome [64]. They subsequently developed two new probes: (1) the mitochondria-targeted WYneN, which exhibited a 10-fold increase in kinetics [65], and (2) a fluorogenic probe, namely CysOx, for live cell imaging [66] (Figure 6B). Similarly, the Carrol team also developed probes for sulfinic acid-modified proteins [67,68], with their latest one used to exhibit higher selectivity over non-oxidized cysteine (DiaAlk, Figure 6B). The application of this probe in different cell lines revealed several interesting findings, such as the fact that the extent of *S*-sulfinylation of certain proteins is cell line-dependent (despite having been exposed to the same concentration of H_2_O_2_) [68].

#### 4.2.3. Protein Carbonylation

Protein carbonylation is a biomarker of oxidative stress, since it is a modification that can take place on various amino acids as well as on the protein backbone. This is because carbonylation can result from three different mechanisms: (1) the direct oxidation of amino acids or protein backbone, (2) the insertion of oxidized lipids (through lipid peroxidation) onto amino acids, and (3) the formation of advanced glycation end products [92]. Due to the reactivity of the carbonyl groups, several probes have been developed over the years. A notable example is the carbonyl-targeted probe developed by Wang and co-workers. As opposed to the commonly used hydrazine as the recognition element, the team developed *m*-APA, an aniline probe with high reaction kinetics and selectivity (Figure 6B) [69]. Chemical proteomics studies in HT1080 cells revealed that during ferroptosis, several carbonylated residues resulted from oxidized lipids insertion (rather by direct oxidation from ROS), and that oxidized voltage-dependent anion-selective channel protein 2 (VDAC2) may be responsible for mediating ferroptosis. Lastly, a highly sensitive carbonyl-target probe for imaging was developed by Sever and co-workers (TFCH, Figure 6B) [70]. The probe was successfully used to monitor protein carbonylation in live cells and in renal tissues during oxidative stress mediated by nephrotoxicity.

## 5. Oxidative Stress in Ageing and Age-Related Neurodegenerative Diseases

In numerous diseases, the amount of ROS is increased and poses a threat to cells and organisms. Oxidative damage has been suggested as both an effect and a mediator of ageing and a range of age-related neurodegenerative pathological conditions [93].

Nutraceuticals treatment of model organisms has remarkably contributed to elucidating the physiological mechanisms associated with biological ageing. For example, data from the nematode *Caenorhabditis elegans* conclude that the effects of polyphenolic phytochemical phlorizin (phloridzin) diminish the intracellular ROS levels (measured by H_2_DCF-DA). This decrease in ROS activates a DAF-16-induced stress response and autophagy, subsequently leading to lifespan extension. Moreover, supplementation with phloridzin reverses the inactivation of dopaminergic neurons observed in worm models of PD [94]. Other compounds, such as the natural product cryptotanshinone [95] or the glucagon-like peptide-1 receptor (GLP-1R) agonist Exendin-4 [96], have been tested in *Caenorhabditis elegans* models of AD, concluding that those drugs can decrease oxidative stress and protein aggregation and improve the disease phenotype. In the following paragraphs we will provide examples of how oxidative stress is implicated in disease manifestations in three age-related neurodegenerative disorders, namely PD, AD and HD.

PD is a progressive age-related neurodegenerative condition associated with a loss of dopaminergic neurons in the substantia nigra pars compacta of the brain. The symptomatology gradually increases upon onset of the disease and is mainly related to motor problems. Although the specific molecular mechanisms underlying the loss of dopaminergic neurons is not fully understood, oxidative stress is a well-established trigger of dopaminergic neurotoxicity [97].

In this line, Soto-Rojas and co-workers determined ROS (through detection of the fluorescence signal of 2,7-dichloro dihydrofluorescein diacetate [DCFH-DA]) and lipid peroxidation (by evaluating the formation of lipid-soluble fluorescent compounds) in the brain of rats with α-synucleinopathy. They found that these two parameters of oxidative stress, as well as mitochondrial complex I dysfunction, positively correlate with neurodegeneration in different brain areas with α-synucleinopathy, such as the substantia nigra pars compacta, the striatum, the hippocampus and the olfactory bulb. Based on in silico studies, they suggested the involvement of peroxisome proliferator-activated receptors (PPAR) alpha (PPAR-α) and gamma (PPAR-γ) in this process [98], since PPARs activation induces fatty acid oxidation (which, in turn, generates ROS) [99]. However, this mechanism needs to be further explored. Two additional recent studies further highlighted the interplay between ROS and PD. Bardien and co-workers sought to understand the pathophysiological mechanisms associated with familiar autosomal dominant PD. More specifically, they studied the p.G849D variant in the neurexin 2α (*NRXN2*) gene, which has been described to co-segregate with PD. Using a cellular model carrying the neurexin 2α p.G849D variant, the authors found elevation of H_2_O_2_ levels (using a commercially available assay), accompanied by neuronal death [100]. Saiki and co-workers wished to shed light on the mechanism that promotes lysosomal retrograde transport, a regulator of autophagy, in PD. The authors initially demonstrated that JIP4 is upstream of the TRPML1-ALG2 pathway, which is known to promote retrograde transport. Subsequently, it was shown that this pathway can be activated in a ROS-dependent manner, since treatment with H_2_O_2_ induces the phosphorylation of JIP4. These results highlight that oxidative stress could, in part, regulate autophagy in the context of PD via the JIP4-TRPML1-ALG2 pathway [101].

AD is a chronic, progressive neurological disorder that is associated with intraneuronal filamentous inclusions, called neurofibrillary tangles, mostly composed by tau protein aggregates, and extracellular senile amyloid plaques, which are predominantly due to the aggregation of misfolded Aβ peptide. Among neurodegenerative diseases, AD is the most common cause of dementia [102]. Similar to PD, alterations in oxidative stress and mitochondrial dynamics and function are known to play a role in the development of the disease [103]. Studies using in vitro models of AD have shown that treatment with the ROS scavenger N-acetyl-L-cysteine (NAC) reduces Aβ deposition through the activation of the PI3K/Akt/GLUT1 pathway, while it ameliorates the impaired glucose uptake phenotype. In this case, the authors measured intracellular ROS levels by flow cytometry [104].

HD is an autosomal dominant neurodegenerative disorder caused by CAG triplet expansions in the *Huntingtin* gene, encoding an elongated poly-glutamine stretch in the Huntingtin protein. Patients with HD exhibit motor, psychiatric and cognitive deterioration and oxidative stress, although it is not clear whether it is the cause or the consequence of disease progression [105]. Ellederová and co-workers found higher oxidative stress in fibroblasts from a minipig model of HD, compared with wild-type minipig fibroblasts. The authors measured ROS (by using CellROX deep red reagent), lipid peroxidation (with Image-iT) and membrane permeability (with Calcein-AM). These elevated levels of oxidative stress correlated with the overexpression of mitochondrial superoxide dismutase 2 (SOD2) and the NEIL3 gene (encoding DNA glycosylase). SOD2 is expressed in the mitochondria, where it controls the cell cycle as a response to oxidative stress, whereas glycosylase enzyme repairs in the replication-associated repair of oxidized DNA. Taken together, these data highlight the role of oxidative stress and DNA damage in HD [106].

Southwell and co-workers overexpressed progerin, a protein that causes premature aging, in murine neurons with HD, and observed ageing-related phenotypes. By treating neurons with H_2_O_2_ and quantifying ROS (with CM-H_2_DCFDA), the authors concluded that biological ageing increases the sensitivity of neurons with HD to exogenous oxidative stress [107,108]. This sensitivity may highlight the interplay between ROS and protein aggregation, as shown for other neurodegenerative diseases.

In the same line, Oliveira and co-workers used MitoParaquat (MitoPQ) to enhance mitochondrial superoxide production. They observed features of PD in MitoPQ-treated zebrafish and an induction of mutant huntingtin aggregation without increasing cell death in a human cell model of HD, upon treatment with MitoPQ [109]. The forkhead box O1 (FOXO1) transcription factor has been shown to be involved in the development of AD and HD. Importantly, upon increased ROS, FOXO1 is activated through the AMPK pathway to promote autophagy, which is a well-established mechanism for the clearance of abnormal proteins and organelles [110].

## 6. Conclusions

When maintained at low levels, ROS serve as signaling molecules and play a positive role in organism survival, by participating in the synthesis of some cellular structures or by being secreted by macrophages as a host defense system against pathogens. Nevertheless, when levels exceed a certain threshold (for instance, due to lower antioxidant defense or higher production), ROS become detrimental to the cell and the organism [111]. The negative consequences of high ROS levels during oxidative stress are due to the oxidative damage to proteins, nucleic acids and lipids, as well as due to aberrant redox signaling [4]. Apart from its documented role in ageing [112], oxidative stress is implicated in a wide variety of age-related diseases such as neurodegenerative disorders [6].

**Figure 7:**
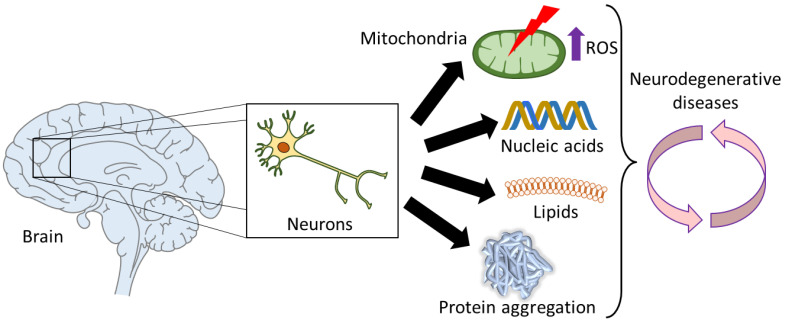
Graphical summary of the association of oxidative stress in neurodegenerative diseases. Neurons in the brain can suffer deleterious modifications due to high levels of ROS. These ROS can be mitochondrial-generated, for instance due to mitochondrial dysfunction, and further contribute to increasing the mitochondrial damage, since ROS can also oxidize DNA, RNA and lipids. Furthermore, protein aggregation is partly caused by ROS, and subsequently, promotes oxidative stress. All these hallmarks of oxidative stress are well-established and may play a role in many age-related neurodegenerative diseases, such as Alzheimer’s, Parkinson’s and Huntington’s diseases.

The prevalence of neurodegenerative disorders is rising and, by 2040, will become the second leading cause of mortality [113]. This group of diseases cause a loss of neurons in the central nervous system and result in defects in memory, movement, cognition or a combination thereof. A common characteristic in many neurodegenerative diseases is the presence of disordered proteins that form toxic aggregates. Several studies have demonstrated the correlation between ROS and protein aggregation for several of these diseases. While it is unclear whether ROS promote aggregation (as in the case of prion protein [8] and α-synuclein [7]) or ROS are produced by aggregates (as in the case of Aβ [15]), these studies certainly place oxidative stress as an important player and call for further insight into its role in neurodegeneration (Figure 7).

Delineating the role of oxidative stress in any condition is not only critical, but also challenging. Due to its importance, several tools for evaluating oxidative stress, including small molecules and genetically encoded protein reporters, have been developed over the years. Perhaps the most commonly used approach is the direct quantification of ROS levels. However, methods that have been traditionally employed need to be used with caution due to the intrinsic properties of ROS (short-lived and similar chemical properties), as was recently highlighted [4]. Herein, we provided an overview of small molecules and protein reporters recently developed to detect different ROS. Apart from exhibiting improved selectivity, several of these tools have additional desirable characteristics, particularly important for in vivo imaging, such as two-photon excitability, ratiometric imaging, organelle-specific localization and others.

While advantageous, the high reaction rates of ROS mean that, unless high concentrations are used, an amount of ROS will evade detection. An approach to bypass this issue is to use methods to define the oxidative damage. Although many oxidative modifications are reversible, they are certainly more stable than free ROS. With regards to proteins, oxidative damage could significantly alter the function and properties of protein. ROS-mediated modifications can take place on eight different amino acid residues as well as on the protein backbone, and this can occur either through direct reaction with ROS or by other oxidized biomolecules (e.g., lipids). Small molecule probes targeting specific oxidized residues have been developed and applied for imaging or protein identification. The latter application (using chemical proteomics) has offered unprecedented information, as in the case of the oxidized pyruvate kinase M2 (which was shown to promote cancer) [88] or the oxidized human prion protein (where aggregation is induced) [8]. The combination of probes with different reactivity (cysteine, sulfenic and sulfinic acids) on *Caenorhabditis elegans* revealed that translation, growth signaling and stress response pathways are redox-sensitive [114].

The emerging role of oxidative stress in neurodegenerative diseases, in combination with the lack of cure for any of these diseases, creates the need for further investigation of this interplay. On the one hand, some evidence suggests that ROS may, to some degree, regulate protein aggregation. On the other hand, protein aggregation seems to be accompanied by an increase in ROS levels, which in turn may impair cell defense mechanisms against protein aggregation (e.g., degradation machinery and heat shock response). In either case, all the above indicate that shedding light on this relationship could provide novel therapeutic approaches in the future. While most methods presented herein were not applied on neurodegenerative diseases, we hope this review will inform the community about these tools and inspire further research to unravel the role of oxidative stress in age-related neurodegenerative pathologies.

## Figures and Tables

**Table 1:** Summary of tools reported in Section 4.

Evaluation Method	Detection	Tool	Application	Key Feature	Notes	Ref
ROS	Hypochlorous acid (HOCl)	FDOCl-1 ^a^	Imaging	Near-infrared (NIR) emission, turn-on probe		[50]
		FDOCl-18 ^a^		Visualization in the off state (additionally to the above)		[51]
		LYSO- & MITO-TP ^a^		Targeted imaging of mitochondria or lysosome		[52]
	Hypobromous acid (HOBr)	RhSN-mito ^a^		Ratiometric probe	Selective over HOCl	[53]
	All hypohalous acids (HOX)	Hypocrates ^b^		First biosensor specific for HOX, ratiometric imaging		[54]
	Superoxide anion (O2^•−^)	DMPS-O ^a^		Turn-on, mitochondria targeting probe with NIR emission		[55]
		HQ ^a^		Two-photon excitability		[56]
		MitoHCy-NH_2_^a^		Detection of ROS generated by a certain protein		[57]
	Peroxynitrite (ONOO^−^)	MITO-CC ^a^		FRET-based, mitochondria targeting probe with two-photon excitability		[58]
		sfGFP(66Thy) ^b^		Turn-on reporters with non-natural amino acids		[59]
		pnGFP-Ultra ^b^				[60]
	Hydrogen peroxide (H_2_O_2_)	Hyp-L ^a^	Imaging/ Proteomics	Labeling of H_2_O_2_-surrounding proteins		[61]
		FROG/B ^b^	Imaging	Excitation state intramolecular proton transfer (ESIPT) mechanism	Requires a single excitation wavelength but its emission is redox-dependent	[62]
Damaged proteins	Oxidized methionine	ReACT ^a^	Proteomics	First selective probe for methionine	Note that results regarding methionine oxidation are by elimination	[63]
	Sulfonic acid	BTD ^a^		High reactivity towards sulfenic acid-modified proteins	Commercial	[64]
		WYneN ^a^		10-fold increase in kinetics (compared to BTD)		[65]
		CysOx ^a^	Imaging	Fluorogenic probe for live cell imaging		[66]
	Sulfinic acid	NO-Bio ^a^	Proteomics	High selectivity over sulfenic acid	Commercial	[67]
		DiaAlk ^a^		Additional selectivity (compared to NO-Bio) over cysteine	Commercial	[68]
	Carbonylation	*m*-APA ^a^		High reaction kinetics and selectivity		[69]
		TFCH ^a^	Imaging	High sensitivity and suitable for live cell imaging		[70]

^a^ Small molecule probe, ^b^ genetically encoded reporter.
